# T‐cell redirecting bispecific antibodies in multiple myeloma: Current landscape and future directions

**DOI:** 10.1002/jha2.729

**Published:** 2023-06-06

**Authors:** Chloe O'Neill, Niels W. C. J. van de Donk

**Affiliations:** ^1^ Amsterdam UMC, Vrije Universiteit Amsterdam Department of Hematology Amsterdam The Netherlands; ^2^ Cancer Center Amsterdam Cancer Biology and Immunology Amsterdam The Netherlands

**Keywords:** BCMA, FcRH5, GPRC5D, immunotherapy, multiple myeloma, T‐cell redirecting bispecific antibody

## Abstract

T‐cell engaging bispecific antibodies (BsAbs) have substantial activity in heavily pretreated patients with multiple myeloma (MM). The overall response rate obtained with B‐cell maturation antigen (BCMA)‐targeting BsAbs is approximately 60%–70%, including a high proportion of patients achieving very good partial response or complete response. Comparable efficacy is seen with BsAbs targeting GPRC5D or FcRH5. Cytokine release syndrome is frequently observed with BsAb treatment, but mostly during the step‐up doses and the first full dose. Early intervention with IL‐6 receptor blocking antibodies (e.g., tocilizumab) prevents escalation to severe manifestations. Infections are also common during treatment and related to the extent of exposure to immune suppressive anti‐MM agents, as well as development of hypogammaglobulinemia due to elimination of normal plasma cells, and probably because of T‐cell exhaustion resulting from continuous BsAb‐mediated T‐cell activation. Adequate monitoring for infections and institution of infectious prophylaxis are essential. Patients treated with GPRC5D‐targteing BsAbs often develop skin and nail disorders and loss of taste, which is likely related to GPRC5D expression in cells that produce hard keratin. Currently ongoing studies are aiming at further improving these results by evaluating BsAbs in combination with other drugs, such as immunomodulatory agents and anti‐CD38 antibodies, as well as the application of BsAbs in earlier lines of therapy, including patients with newly diagnosed disease. We expect that the outcomes of patients with MM will further improve by the introduction of this novel type of T‐cell immunotherapy.

## 1 CASE PRESENTATION

In March 2019, at the age of 64 years, one of our patients with multiple myeloma (MM), experienced for the 11th time disease progression with development of new bone lesions. At that time his disease was refractory to three immunomodulatory drugs (IMiDs; namely thalidomide, lenalidomide, and pomalidomide), one CelMOD (cereblon modifying drug; iberdomide), two proteasome inhibitors (PIs; bortezomib and carfilzomib), one CD38‐targeting antibody (daratumumab), as well as alkylating drugs (refractory to cyclophosphamide [and exposed to melphalan]) and one checkpoint inhibitor (the anti‐PD‐L1 blocking antibody durvalumab). Cytogenetic analysis was repeated and revealed the presence of a new chromosome 17p deletion, next to the preexisting hyperdiploidy.

## 2 WHAT IS THE PROGNOSIS OF HEAVILY PRETREATED, TRIPLE‐CLASS REFRACTORY MYELOMA PATIENTS?

At the time of his 11th relapse, our patient was heavily pretreated and refractory to three important drug classes (IMiDs, PIs, and CD38‐targeting antibodies). These triple‐class refractory MM patients have a very poor prognosis [1]. The prospective LocoMMotion study, which studied clinical outcomes of triple‐class exposed MM patients, who were treated outside of clinical trials, showed that there is currently no standard‐of‐care treatment for triple‐class refractory MM patients, because 92 unique treatment regimens were used to treat 248 triple‐class exposed patients (183 of these 248 patients were triple‐class refractory at baseline) [2]. Patients who were triple‐class refractory had an overall response rate of 25.1% and only a median progression‐free survival (PFS) of 3.9 months and median overall survival (OS) of 11.1 months [2]. Comparable poor survival was seen in the retrospective MAMMOTH study with a median OS of 5.3 months for patients who were refractory to lenalidomide, pomalidomide, bortezomib, carfilzomib, and a CD38‐targeting antibody (penta‐drug refractory disease) [3].

## 3 HOW TO TREAT TRIPLE‐CLASS REFRACTORY PATIENTS?

**FIGURE 1:**
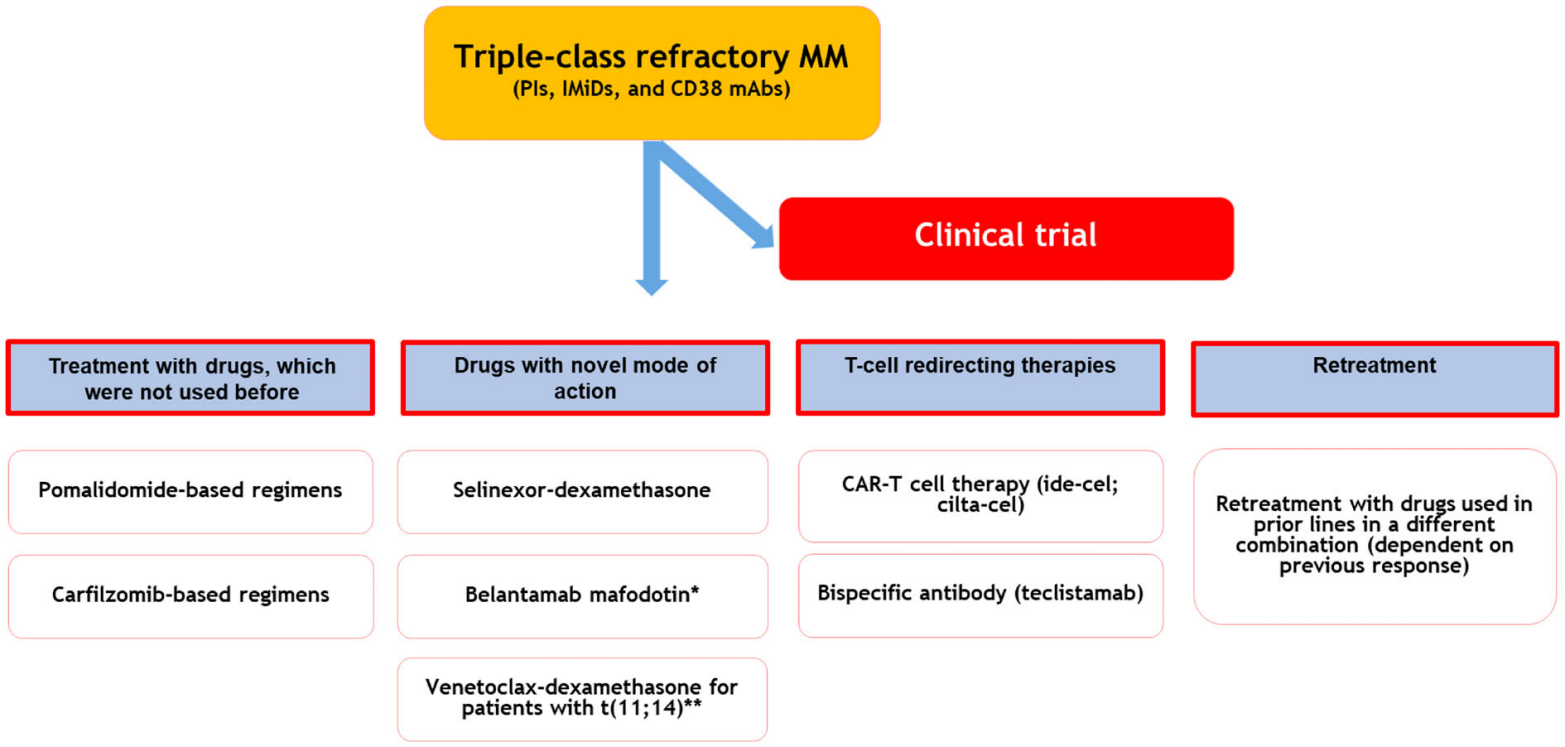
Treatment options for triple class‐refractory patients. Treatment choice is dependent on patient‐characteristics (e.g., comorbidities, age, preferences), tumor‐related features (e.g., tumor growth kinetics, presence of extramedullary disease), and previous treatment that the patient received, as well as local availability of drugs and reimbursement issues. *Because the confirmatory phase 3 DREAMM‐3 study did not show a PFS advantage of belantamab mafodotin over pomalidomide‐dexamethasone, belantamab mafodotin was withdrawn from the US market in November 2022. **Not yet approved; additional data will be provided by the ongoing CANOVA study, which evaluates venetoclax plus dexamethasone versus pomalidomide‐dexamethasone in patients with t(11;14)‐positive RRMM.

Triple‐class refractory patients should always be considered for participation in a clinical trial, which allows early access to novel agents with new mechanisms of action (Figure 1). Unfortunately, trial participation is frequently not possible due to presence of an aggressive relapse and need to directly start treatment, or because of ineligibility for trial participation (e.g., not fulfilling the inclusion and exclusion criteria due to presence of non‐secretory disease, thrombocytopenia, or impaired creatinine clearance). In case patients were not previously treated with pomalidomide or carfilzomib, regimens including these drugs are a good option. Alternatively, these patients can be treated with drugs used in prior lines in a different, potentially synergistic combination (Figure 1). Conventional chemotherapy regimens, such as DCEP (dexamethasone, cyclophosphamide, etoposide, and cisplatin) or DT‐PACE (dexamethasone, thalidomide, cisplatin, doxorubicin, cyclophosphamide, and etoposide) can be useful in patients with high tumor burden or extramedullary disease as a bridge towards another therapy such as chimeric antigen receptor (CAR) T‐cell therapy or a clinical study [4]. In addition, based on local availability and reimbursement status, treatment with recently approved drugs with a novel mode of action, such as selinexor and belantamab mafodotin, can be considered [5]. Selinexor inhibits XPO1‐mediated nuclear export resulting in the nuclear accumulation and activation of tumor suppressor proteins. In combination with dexamethasone, selinexor induced at least partial response (PR) in 26% of triple‐class refractory patients with a median PFS of 3.7 months [6]. Nausea, anorexia, diarrhea, hyponatremia, thrombocytopenia, and fatigue led to frequent treatment interruptions and dose reductions [6]. Combination studies are ongoing, including studies with weekly administration or using a lower dose of selinexor to reduce toxicities. Belantamab mafodotin is a B‐cell maturation antigen (BCMA)‐targeting antibody linked to a microtubule inhibitor (monomethyl auristatin F [MMAF]) via a protease‐resistant maleimidocaproyl linker. In the DREAMM‐2 study belantamab mafodotin at the dose of 2.5 mg/kg induced at least PR in 31% of patients with a median PFS of 2.9 months [7]. The most common grade 3–4 adverse events were keratopathy (27% in the 2.5 mg/kg cohort). Because the confirmatory phase 3 DREAMM‐3 study did not show a PFS advantage of belantamab mafodotin over pomalidomide‐dexamethasone, belantamab mafodotin was withdrawn from the US market in November 2022. Combination studies with belantamab mafodotin are ongoing, including combinations with pomalidomide‐dexamethasone, bortezomib‐dexamethasone, and novel agents (such as an OX40 agonist or PD‐1 blocker) [8]. Although not yet approved, venetoclax has substantial activity in patients carrying the t(11;14) [9]. Additional data will be provided by the ongoing CANOVA study, which evaluates venetoclax plus dexamethasone versus pomalidomide‐dexamethasone in patients with t(11;14)‐positive RRMM.

Other approved options include BCMA‐targeting CAR T‐cell therapies (ide‐cel and cilta‐cel). Ide‐cel received European Medicines Agency (EMA) and U.S. Food and Drug Administration (FDA) approval based on an overall response rate of 73% and median PFS of 8.8 months in patients with a median of 6 prior lines of therapy [10]. Cilta‐cel is able to bind to BCMA with two binding domains leading to high affinity interaction with the tumor cells, and this may explain the superior activity, compared to what is achieved with ide‐cel [11]. In a comparable patient population (median of six prior lines of therapy) cilta‐cel induced at least PR in 98% of patients with at 27 months 55% of the patients free of progression and alive [12, 13]. Most common toxicities observed with CAR T‐cell therapy include cytokine‐release syndrome and cytopenias. Although CAR T‐cell therapy has as substantial advantage that it only needs to be administered once (patients benefit from treatment‐free interval), the manufacturing time, which may be as long as 6–8 weeks, precludes the treatment of patients with rapidly evolving disease. More recently, patients with advanced MM can also be treated with T‐cell redirecting bispecific antibodies (BsAb) following the approval of the off‐the‐shelf available T‐cell engaging BsAb teclistamab, which binds to both BCMA on the tumor cells and CD3 on T cells (for details next sections).

## 4 CASE CONTINUED 1

In March 2019, we enrolled our triple‐class refractory patient into the phase 1, dose‐escalation trial with teclistamab (MajesTEC‐1). At that time he received only a fraction of the recommended phase 2 dose (RP2D), which was identified later. Prior to the priming dose and first full dose he received premedication (dexamethasone, anti‐histamine, and acetaminophen) to prevent severe cytokine release syndrome (CRS). Nonetheless, 1 h following the administration of the first priming dose, he developed a fever (38.7^◦^C) in the absence of signs of infection. His blood pressure was normal, but his heart rate was increased (100 beats per minute). He was not hypoxic. The diagnosis of grade 1 CRS was made, and he was treated with one intravenous (IV) infusion of tocilizumab. The fever rapidly resolved, and we could continue treatment with teclistamab as planned. As infectious prophylaxis he received valacyclovir, cotrimoxazole, and we started IVIG treatment when he developed hypogammaglobulinemia. Although heavily pretreated he rapidly achieved a deep remission with as best response a stringent complete response (CR).

## 5 HOW DO T‐CELL ENGAGING BISPECIFIC ANTIBODIES WORK?

**FIGURE 2:**
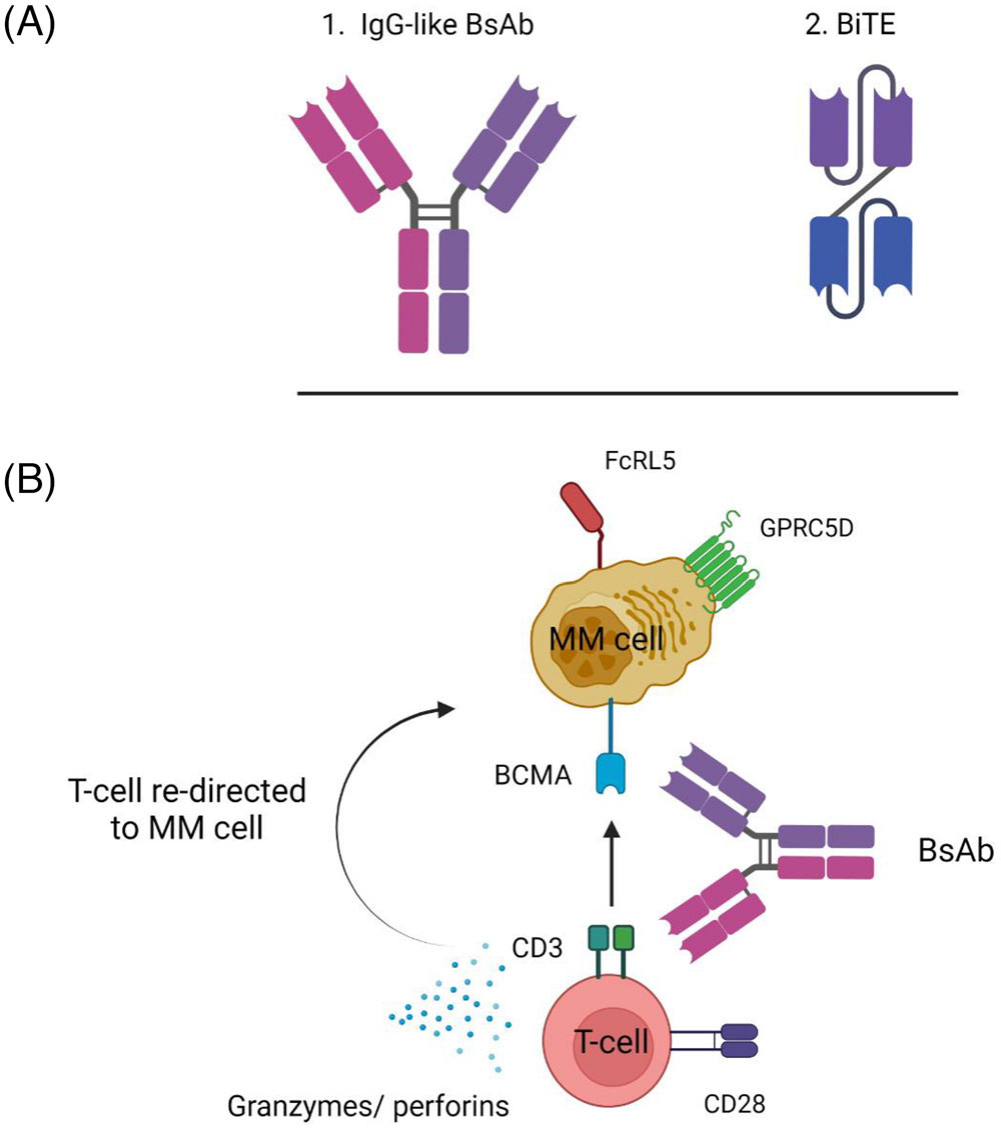
Structure and mode of action of T‐cell redirecting bispecific antibodies. (A) Structure of an IgG‐like bispecific antibody and a BiTE molecule. (B) A T‐cell redirecting bispecific antibody simultaneously binds to a tumor‐associated antigen, B‐cell maturation antigen (BCMA) in this case, and to CD3 present on the T‐cell surface. This results in the redirection of T‐cells to multiple myeloma (MM) cells, and the subsequent activation and degranulation of T‐cells. The cyotoxic granzyme B/perforin pathway is used by the T‐cell to induce MM cell death.

BsAbs consist of two different binding domains, which interact either with CD3 on T‐cells or a tumor‐associated antigen (TAA) on the tumor cell surface (Figure 2). This redirects the T‐cell to the tumor cell and enables the formation of an immunological synapse. In this way, the T cell is activated and can carry out effector functions, such as cytokine production and degranulation without the need for antigen presenting cells or major histocompatibility complex class I or class II molecules [14]. The TAA of choice should be homogenously expressed by tumor cells and not expressed by healthy tissues [14]. Targeting a TAA that is essential for the tumor cell is favorable, so that downregulation or loss of the antigen would be detrimental to the tumor [14]. Commonly targeted antigens of BsAbs for MM treatment are BCMA, GPRC5D, and FcRH5.

BsAbs come in many different formats but can generally be classified into two groups; IgG‐like BsAbs containing a fragment crystallizable (Fc) domain and those without an Fc domain (e.g. BiTEs and DARTs) (Figure 2). The Fc domain of IgG‐like BsAbs prolongs the elimination half‐life by increasing the molecular size of the molecule, and by enabling binding to the neonatal Fc receptor (FcRn), which recycles IgG [15]. The prolonged half‐life makes intermittent administration of IgG‐like BsAb possible, which is convenient for patients and caregivers. The Fc tail is silenced for other effector functions, including binding to complement component C1q and to FcγRs to prevent aspecific T‐cell activation. BsAbs without a Fc tail, such as BiTEs, have a short half‐life, making continuous IV administration necessary.

Trispecific antibodies are also under investigation in both preclinical studies and in clinical trials. Next to binding CD3, these antibodies either target two antigens on the myeloma cell in order to prevent antigen escape, or they have the ability to activate T‐cell co‐stimulatory molecules to prevent T‐cell anergy [16, 17].

## 6 WHICH TARGETS ARE COMMONLY USED IN MULTIPLE MYELOMA?

### 6.1 B cell maturation antigen

B cell maturation antigen (BCMA) is a type III transmembrane glycoprotein that belongs to the tumor necrosis family receptors. It is expressed on healthy and malignant plasma cells and a subset of mature B cells [18]. It promotes the proliferation and survival of B cells by interacting with its ligands B cell activating factor and a proliferation‐inducing ligand (APRIL) [19]. Its downstream effects lead to the activation of NF‐κB, ETS‐like transcription factor 1 (Elk‐1), c‐jun N‐terminal kinase, and p38, resulting in increased expression of anti‐apoptotic molecules such as Bcl‐2 and Bcl‐XL which support plasma cell survival [20]. Elevated levels of soluble BCMA (sBCMA) are found in the serum of MM patients, correlating with disease burden [21, 22, 23]. sBCMA is produced by γ‐secretase, which cleaves BCMA from the cell membrane [24].

### 6.2 G Protein‐Coupled Receptor Family C Group 5 Member D (GPRC5D)

GPRC5D is an orphan G‐protein coupled receptor. It is a 7‐segment transmembrane protein for which the endogenous ligand(s) and signaling mechanisms remain unknown [25, 26]. GPRC5D is expressed by malignant plasma cells and also, albeit to a lesser extent, on normal plasma cells [25]. In addition, GPRC5D is expressed by cells that produce hard keratin, such as cortical cells of the hair shaft, the keratogenous zone of the nail, and filiform papillae of the tongue [26, 27, 28]. GPRC5D expression has also been found in the inferior olivary nucleus, a structure located in the medulla oblongata that relays motor and sensory signals from the spinal cord to the cerebellum and regulates motor coordination. This may explain why a subset of patients treated with GPRC5D‐targeting CAR T‐cell therapy developed neurotoxicity with cerebellar symptoms [29].

### 6.3 Fc Receptor‐Homolog 5 (FcRH5)

FcRH5 is a membrane surface protein that is related to the group of receptors homologous to FcγRI [30], [31]. It is solely expressed in the B cell lineage where it is first expressed in pre‐B cells and increases in expression through the maturation process to plasma cells [32]. The restriction of FcRH5 expression to B cells and its elevated expression in MM cells makes it an attractive target.

## 7 CLINICAL ACTIVITY OF BCMA‐TARGETING T‐CELL ENGAGING BISPECIFIC ANTIBODIES IN MM

Proof‐of‐concept that T‐cell engaging bispecific antibodies work in heavily pretreated MM patients was delivered by the clinical study that evaluated AMG 420. This BiTE molecule binds to both BCMA on MM cells and CD3 on T‐cells. AMG 420 has a short half‐life and therefore needs to be administered via continuous IV infusion (every 4 weeks of a 6‐week cycle) [33]. At the maximum tolerated dose of 400 μg/day the overall response rate was 70% [33]. Responses occurred early during the first treatment cycle, and were durable with some responses lasting >1 year [33]. Although AMG 420 has substantial activity, the continuous IV infusion is cumbersome for patients and may also lead to intravascular catheter‐related infections. Therefore new T‐cell engaging antibody formats were developed with longer half‐life, enabling intermittent administration. The IgG‐like BsAb teclistamab is the first approved BsAb in MM. In 2022 it received EMA approval for the treatment of adult patients with relapsed and refractory multiple myeloma, who have received at least three prior therapies, including an IMiD, a PI and an anti‐CD38 antibody, and FDA approval to treat patients who have received at least four prior lines of therapy, including a PI, an IMiD, and an anti‐CD38 monoclonal antibody.

In the first‐in‐human, dose‐escalation study, the recommended phase 2 dose (RP2D) of teclistamab was established as a subcutaneous (SC) dose of 1.5 mg/kg with 2 step‐up doses to mitigate CRS (0.06 mg/kg and 0.3 mg/kg) [34]. SC dosing is frequently more comfortable for patients than IV administration and is typically also faster which may be of benefit for the organization of the outpatient clinic. The dose‐escalation was followed by an expansion cohort with an additional 125 patients treated at the RP2D. In total 165 patients (78% with triple‐class refractory disease) received teclistamab at the RP2D with at least PR in 63% and CR in 39% of the patients (Table 1) [35]. The median PFS was 11.3 months, and median response duration was 18.4 months [35]. Because the MajesTEC‐1 study did not contain a control arm, an indirect treatment comparison was conducted with patients from the prospective, real‐world LocoMMotion study [36]. Patients treated with teclistamab were 2.3 times more likely to reach at least PR and 5.2 times more likely to reach very good PR (VGPR) or better compared to patients from the LocoMMotion study. Also response duration and PFS were significantly better with teclistamab compared to real‐world physician's choice of therapy in the LocoMMotion study [36]. Results with teclistamab also compared favorably to that obtained with selinexor‐dexamethasone (STORM study) or belantamab mafodotin (DREAMM‐2 study) [36, 37]. Teclistamab was also evaluated in patients with prior exposure to another BCMA‐targeted therapy (CAR T‐cell therapy or antibody‐drug conjugate) [38]. There was no difference in safety profile, compared to patients without prior BCMA‐targeted therapy. At least PR was achieved by 55.2% of patients previously exposed to an antibody‐drug conjugate and 53.3% in patients who were previously treated with CAR T‐cells [38].

**TABLE 1:** Studies evaluating T‐cell redirecting BsAbs as monotherapy in heavily pretreated MM patients.

	Teclistamab [35]	Elranatmab [39]	Linvoseltamab (REGN5458) [44]	ABBV‐383 (TNB‐383B) [43]	Alnuctamab (CC‐93269) [40]	Talquetamab [48]	Forimtamig (RG6234) [49]	Cevostamab [50]
**Target**	BCMA	BCMA	BCMA	BCMA	BCMA	GPRC5D	GPRC5D	FcRH5
***N***	165 patients treated at the RP2D	123 patients treated at the RP2D	73	124; 81 treated with ≥40 mg IV Q3W	68	Two RP2Ds: 143 patients treated at the 400 μg/kg QW dosing schedule and 145 patients treated at the 800 μg/kg Q2W dosing schedule	IV arm: 51 SC arm: 57	161
**PR (%)**	63.0	61.0	200–800 mg: 75	≥40 mg: 68.4	53	400 μg/kg: 74.1 800 μg/kg: 73.1	IV arm: 71.4 SC arm: 63.6	132–198 mg: 56.7
**CR (%)**	39.4	27.6	200–800 mg: 16	≥40 mg: 36.7	23	400 μg/kg: 33.6 800 μg/kg: 32.4	IV arm: 34.7 SC arm:25.5	132–198 mg: 8.4
**Median PFS (months)**	11.3	Not reached; 12‐months PFS rate: 58.8%	NR	≥40 mg: not reached; 12‐month PFS rate was 57.9%	NR	400 μg/kg: 7.5 months 800 μg/kg: 11.9 months	NR	NR
**CRS, all grade (%)**	72.1	56.3	3‐800 mg: 38.4	≥40 mg: 72.8	53	400 μg/kg: 79.0 800 μg/kg: 72.4	IV arm: 82.4 SC arm: 78.9	80.7
**CRS, ≥grade 3 (%)**	0.6	0.0	3‐800 mg: 0.0	≥40 mg: 3.7	0	400 μg/kg: 2.1 800 μg/kg: 0.7	IV arm: 2.0 SC arm: 1.8	37.9
**Neurotoxicity, all grade (%)**	14.5 (ICANS: 3.0)	ICANS: 3.4	3–800 mg: NR; (ICANS: 4.1)	NR	3	ICANS 400 μg/kg: 10.7 ICANS 800 μg/kg: 10.1	ICANS IV arm: 9.8 ICANS SC arm: 12.3	ICANS: 14.3

Abbreviations: BCMA, B‐cell maturation antigen; ICANS, immune effector cell‐associated neurotoxicity syndrome; NR, not reported; RP2D, recommended phase 2 dose.

Several other IgG‐like BCMA‐targeting antibodies are in development for the treatment of MM, including elranatamab (Table 1). Elranatamab was administered via SC injections to 123 patients at the RP2D of 76 mg, preceded by 2 step‐up doses (12 and 32 mg) [39]. These patients were heavily pretreated with 96.7% being triple‐class refractory [39]. With a follow‐up of 10.4 months, the overall response rate was 61.0%, and the 12‐month PFS rate was 58.8% [39].

Other IgG‐like BCMA‐targeting BsAbs in clinical development are alnuctamab (CC‐93269) [40], HPN217 [41], WVT078 [42], ABBV‐383 [43], and REGN‐5458 [44] (Table 1). Development of the half‐life extended BiTE AMG 701, which was developed to replace AMG 420, was recently halted [45]. All these BsAbs are characterized by high antitumor activity in heavily pretreated patients and a manageable toxicity profile with CRS, cytopenias, and infections as the most common adverse events. However, there are differences between these antibodies. This includes the way of administration (IV vs. SC), frequency of administration (every week, or every 3 weeks after treatment initiation), and need for step‐up dosing (see also below).

## 8 CLINICAL ACTIVITY OF OTHER T‐CELL ENGAGING BISPECIFIC ANTIBODIES IN MM

Not only BsAbs targeting BCMA have shown great promise, but also BsAbs targeting other surface antigens on MM cells have substantial anti‐MM activity (Table 1). Importantly sequential use of different T‐cell immunotherapies is possible and can induce deep and durable remissions [46].

## 9 GPRC5D‐TARGETING BSABS

Talquetamab is the first‐in‐class GPRC5D‐targteing BsAb, which was tested in a first‐in‐man, dose‐escalation study in heavily pretreated MM patients (median of six prior lines of therapy)[47], [48]. This study identified two RP2Ds: 400 μg/kg SC every week and 800 μg/kg SC every other week [47]. Although heavily pretreated (74.1% triple‐class refractory) at least PR was achieved in 74.1% of patients treated at the 400 μg/kg dose, including CR in 33.6%. Comparable response rates were seen in patients treated at the 800 μg/kg dose (69.0% triple‐class refractory) with at least PR in 73.1% and CR in 32.4% [48]. The median PFS was 7.5 months at the 400 μg/kg dose and 11.9 months at the 800 μg/kg dose. In patients with previous exposure to BCMA‐targeting agents at least PR was achieved in 62.7% [48].

Forimtamig (RG6234) is another GPRC5D T‐cell engaging BsAb with a 2:1 (GPRC5D:CD3) configuration, which increased the potency compared to the 1:1 configuration [49]. Forimtamig induced rapid responses in heavily pretreated MM patients (≥PR with IV administration: 71.4%; ≥PR with SC administration: 63.6%) [49]. The toxicity profile of forimtamig is comparable to that observed with talquetamab [49].

## 10 FCRH5‐TARGETING BSABS

Cevostamab is a BsAb that targets the membrane‐proximal region of FcRH5 on the MM cell surface. The first‐in‐human dose‐escalation study with cevostamab has shown promising activity in heavily pretreated patients [50]. At higher dose levels (132‐198 mg), at least PR was achieved by 56.7% including CR in 8.4%. In the single step‐up cohorts the median duration of response was 11.5 months [50]. Cevostamab has a manageable toxicity profile with as most common adverse events CRS, cytopenias, and infections [50].

## 11 WHAT IS THE ADVERSE EVENT PROFILE OF A T‐CELL REDIRECTING BISPECIFIC ANTIBODY?

### 11.1 Cytokine‐release syndrome

**FIGURE 3:**
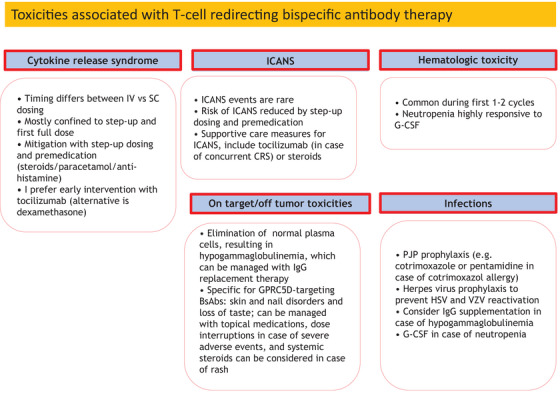
Management of the different adverse events associated with T‐cell redirecting BsAb treatment. IV, intravenous; SC, subcutaneous; ICANS, immune effector cell‐associated neurotoxicity syndrome; PJP, pneumocystis Jirovecii pneumonia; HSV, herpes simplex virus; VZV, varicella zoster virus; G‐CSF, granulocyte colony‐stimulating factor.

Cytokine‐release syndrome is a frequent manifestation following initiation of BsAb treatment, and mostly observed during the step‐up doses or the first full dose (Figure 3). BsAb‐mediated T‐cell activation leads to the production of inflammatory cytokines, such as IL‐6 and tumor necrosis factor‐α, which may induce a systemic inflammatory response with fever, low blood pressure, and sometimes hypoxia.

Development of severe CRS can be prevented by premedication including steroids (typically administered during step‐up dosing and first full dose). Also the strategy of starting with a low dose of the BsAb, allows for the safe subsequent administration of a higher dose. Some BsAbs use two‐step doses, as a 2‐step‐up dosing regimen appeared to further mitigate severity of CRS, compared to only one step‐up dose [50], [51]. Interestingly, ABBV‐383 can be administered without the need for step‐up dosing. Also the hospitalization requirement for this agent is therefore relatively short (first 48 h after initial administration) [43]. These properties of ABBV‐383 are likely related to the low affinity binding to CD3 on the T‐cells, while BCMA‐binding occurs with high affinity, which also translated into antitumor activity with minimal cytokine release in preclinical studies [52]. Administration of the IL‐6 receptor blocking antibody, tocilizumab, prior to the first dose of cevostamab significantly reduced the CRS rate [53]. There was no negative effect of tocilizumab on efficacy of cevostamab, but there was a higher rate of neutropenia [53]. Tocilizumab as CRS prophylaxis has the potential to enable outpatient administration of BsAbs.

When CRS occurs it can be effectively treated by using tocilizumab, or steroids. We typically administer tocilizumab directly when a patient develops grade 1 CRS in order to prevent further escalation to more severe manifestations. Other supportive care measures include acetaminophen in case of fever, IV fluids in case of hypotension, and O2 supplementation in case of hypoxia. Intensive care treatment with vasopressor support may be needed in patients who develop high‐grade CRS.

### 11.2 On target/off tumor toxicities

For a protein to be an appropriate target for BsAbs, the protein must be uniformly expressed on the cell surface of the tumor cells, but not on critical normal tissues to prevent severe “on target, off tumor” toxicities (Figure 3).

Because BCMA, GPRC5D and FcRH5 are also expressed on normal plasma cells, these antibody‐producing cells are also eliminated by BsAbs. This results in hypogammaglobulinemia, which increases risk for development of infections [25]. Interestingly, GPRC5D expression is lower and CD38 expression is higher on normal PCs, when compared to MM cells, while BCMA and FcRH5 expression are similar [25], [32], [54]. These differences in target expression may translate into different frequencies of hypogammaglobulinemia. Immunoglobulin replacement therapy can be considered in patients who develop hypogammaglobulinemia to prevent infections.

GPRC5D is also expressed on cells that produce keratin, which may explain the development of skin and nail disorders in patients treated with GPRC5D‐targeting BsAbs. These adverse events are typically of low grade [55, 56, 57]. Patients may also develop oral toxicities, including loss of taste, dry mouth, and dysphagia [55, 56, 57, 58]. A single‐centre study showed that these GPRC5D‐specific adverse events can be managed with topical medications (e.g., triamcinolone cream and emollients for skin toxicities; saliva substitute sprays and rinses for oral toxicities), while dose interruptions are only needed in case of severe adverse events. In addition, rash can be effectively treated with systemic steroids in conjunction with topical medications [58]. Several studies are ongoing to investigate novel strategies to mitigate these “on target, off tumor” side effects. Skin and nail disorders were also frequently observed after GPRC5D‐targeting CAR T‐cell therapy, but taste changes were less frequent (6%) [29], [59], [60].

### 11.3 Cytopenias

Especially during the first 1 or 2 cycles patients may develop neutropenia, thrombocytopenia and anemia (Figure 3). This is probably caused by the local production of cytokines in the bone marrow, which may temporarily suppress normal hematopoiesis. These effects are likely more pronounced in these late‐line patients, as they have received large amounts of myelotoxic compounds in previous lines of therapy (e.g., IMiDs and alkylating drugs). Neutropenia can be easily managed by administration of granulocyte colony‐stimulating factor.

### 11.4 Infections

Patients treated with BsAbs have a high frequency of infections which may be related to (1) the high cumulative exposure to immunosuppressive drugs during previous lines of therapy, (2) development of hypogammaglobulinemia due to eradication of normal plasma cells, (3) occurrence of neutropenia, and (4) the development of T‐cell exhaustion during long‐term BsAb treatment (Figure 3) [61], [62]. In a pooled analysis, non‐BCMA targeted BsAbs were associated with lower grade 3/4 infections when compared to BCMA‐targeted bispecific antibodies [63].

Because of the immunocompromised status of these heavily pretreated patients, there should be attention and close monitoring for infections, including those caused by opportunistic pathogens, like pneumocystis jirovecii pneumonia (PJP), or toxoplasma gondii. Reactivation of viral infections may also occur during treatment with a BsAb, including cytomegalovirus, herpes simplex virus, herpes zoster virus, BK virus, or hepatitis B virus.

In our practice, we give patients treated with a BsAb infectious prophylaxis consisting of valacyclovir to prevent herpes simplex virus and varicella zoster virus‐related infections, as well as PJP prophylaxis with cotrimoxazol or another drug in case of allergy (e.g., pentamidine). Patients with increased risk for infections may also benefit from levofloxacin to prevent bacterial infections (e.g., first two cycles) [64]. We start immunoglobulin replacement therapy in the presence of hypogammaglobulinemia (IgG < 4 g/L).

## 12 CASE CONTINUED 2

Approximately 4.0 years after start of teclistamab, he is still treated with this agent (once montly), and he remains in a stringent complete remission. He has in fact achieved the deepest and most durable remission, since he was diagnosed with MM. We continued cotrimoxazol and valacyclovir as well as immunoglobulin replacement therapy, because his IgG levels did not recover during treatment. With this supportive care cocktail, he did not develop severe infections, or other complications.

## 13 WHAT ARE RESISTANCE MECHANISMS TO BISPECIFIC ANTIBODIES?

Our patient has a very durable remission, but not all patients respond (primary resistance) and patients may develop disease progression after having achieved a remission (acquired resistance). Both T‐cell‐related features, tumor characteristics, and components of the bone marrow microenvironment may contribute to resistance to T‐cell immunotherapies [65]. Several studies have shown that high tumor burden and presence of extramedullary disease is associated with an inferior response to BsAbs [35], [39]. In addition, a high number of regulatory T‐cells or high proportion of exhausted T‐cells is associated with failure to respond to BsAb treatment [25], [66], [67]. Disease progression may be related to loss of target antigen expression or development of T‐cell exhaustion due to continuous BsAb‐mediated T‐cell activation [62], [68].

## 14 HOW TO FURTHER BUILT ON THESE RESULTS?

### 14.1 Combination strategies

Although BsAbs have shown promising activity in heavily pretreated patients, a subset of patients does not respond and eventually many responding patients relapse. Aiming at further improving depth and duration of response, several combination strategies are being investigated. This includes combinations with agents that improve T‐cell function, such as IMiDs (e.g., lenalidomide and pomalidomide), CelMODs (e.g., iberdomide), checkpoint inhibitors (e.g., antibodies inhibiting the PD‐1/PD‐L1 axis), as well as CD38‐targeting antibodies [18], [69], [70].

We have shown that T‐cells obtained from daratumumab‐exposed patients are better effector cells in the presence of a BsAb, compared to T‐cells from daratumumab‐naïve patients [18]. This may be related to the daratumumab‐induced elimination of CD38^+^ regulatory T‐cells leading to improved T‐cell numbers and killing capacity [71], [72]. Altogether this formed the preclinical rationale for ongoing combination studies with daratumumab and a BsAb. Preliminary evidence shows that the combination of daratumumab plus teclistamab or talquetamab has a promising activity in patients, who were mostly refractory to anti‐CD38 antibody‐based therapy. Talquetamab (at a dose of 400 μg/kg every week or 800 μg/kg every other week) plus daratumumab induced at least PR in 80.4% of 51 response‐evaluable patients (48.3% of patients was exposed to BCMA‐targeted therapy and 75.9% was anti‐CD38 antibody refractory) including VGPR or better in 62.7% and CR in 29.4% [56]. Among 51 response‐evaluable patients (12.3% exposed to BCMA‐targeted therapy, and 63.1% anti‐CD38 antibody refractory), teclistamab in combination with daratumumab induced at least PR in 76.5% of the patients including VGPR or better in 70.6% and CR in 21.6% [73]. With both combinations higher response rates were observed in patients with anti‐CD38 antibody sensitive disease. Serious infections are common in patients treated with BsAbs, with probably a higher rate of such infections with BCMA‐targeting BsAbs compared to BsAbs targeting other MM surface antigens [35], [47], [63]. The incidence and severity of infections may also increase with combination therapy, because combination partners not only have anti‐MM activity, but may also have a negative impact on normal immune cells [74]. For example, daratumumab eliminates natural killer cells and normal plasma cells [75, 76, 77], and therefore daratumumab‐based combination strategies may increase the rate of serious infections.

In an attempt to prevent antigen escape, there is also an ongoing study assessing the combination of two BsAbs targeting two different MM‐associated antigens (RedirecTT‐1: combination of teclistamab plus talquetamab). Based on preclinical data showing that gamma‐secretase inhibitors are capable of increasing BCMA expression on the tumor cell surface and concomitantly reducing soluble BCMA levels, which resulted in enhanced activity of BCMA‐targeting BsAbs [78], BCMA‐targeting BsAbs are also evaluated in combination with gamma‐secretase inhibitors in clinical trials.

### 14.2 Different treatment schedules

Fixed treatment duration and less frequent administration are also explored as novel strategies to prevent BsAb‐mediated T‐cell exhaustion and thereby reduce the frequency of infections and/or improve the durability of response. Recently preclinical studies showed that treatment‐free intervals may also be beneficial to preserve T‐cell fitness and improve anti‐tumor activity [62].

### 14.3 Earlier lines of therapy

Patients with newly diagnosed disease or early relapsed/refractory disease have superior T‐cell function and numbers when compared to end‐stage patients who received immunosuppressive anti‐MM drugs over a period of many years [79]. Therefore BsAbs are currently evaluated in earlier lines of therapy, including newly diagnosed patients. Examples of studies evaluating BsAbs in newly diagnosed disease are the MajesTEC‐7 study, in which patients who are either ineligible or not intended for autologous stem cell transplantation are randomized to either teclistamab plus daratumumab and lenalidomide (experimental arm) or to daratumumab‐lenalidomide‐dexamethasone (control arm). Phase 3 studies are also evaluating the use of BCMA‐targeting BsAbs as novel maintenance therapy post‐transplant (MajesTEC‐4: teclistamab vs. teclistamab plus lenalidomide vs. lenalidomide; MagnetisMM‐7: elranatamab vs. lenalidomide).

## 15 CONCLUSIONS

In conclusion, T‐cell immunotherapy with BsAbs has demonstrated substantial activity in heavily pretreated, triple‐class refractory MM patients. CRS and neurotoxicity are typically less severe with BsAbs than with CAR T‐cell therapy, indicating that also elderly patients can be effectively treated with this new class of agents. Ongoing efforts are exploring combination strategies and earlier use of BsAbs, including its evaluation in newly diagnosed disease. Altogether these efforts will lead to a further improvement of the survival of MM patients.

## AUTHOR CONTRIBUTIONS

Chloe O'Neill and Niels W.C.J. van de Donk contributed to the writing of the manuscript and literature search.

## CONFLICT OF INTEREST STATEMENT

C.O.N. has no conflict of interest. N.W.C.J.v.d.D. has received research support from Janssen Pharmaceuticals, AMGEN, Celgene, Novartis, Cellectis, and BMS and serves in advisory boards for Janssen Pharmaceuticals, AMGEN, Celgene, BMS, Takeda, Roche, Novartis, Bayer, Adaptive, and Servier
